# Age Estimation by Dental Calcification Stages and Hand-Wrist Radiograph

**DOI:** 10.7759/cureus.29045

**Published:** 2022-09-11

**Authors:** Sujata Kumari, Amit Kumar Sahu, Jagdish Rajguru, Pooja Bishnoi, Aseem Jolly Garg, Raksha Thakur

**Affiliations:** 1 Pediatric Dentistry, Dr. R. Ahmed Dental College and Hospital, Kolkata, IND; 2 Pedodontics and Preventive Dentistry, Shyam Shah Medical College and Associated Hospital, Rewa, IND; 3 Oral and Maxillofacial Pathology, Hi-Tech Dental College and Hospital, Bhubaneswar, IND; 4 Pediatric Dentistry, Vyas Dental College & Hospital, Jodhpur, IND; 5 Conservative Dentistry and Endodontics, Adesh Institute of Dental Sciences and Research, Bathinda, IND; 6 Pedodontics and Preventive Dentistry, Guru Gobind Singh College of Dental Sciences and Research Centre, Burhanpur, IND

**Keywords:** demirjian, skeletal, hand-wrist radiograph, dental calcification, age estimation

## Abstract

Introduction: An essential part of pediatric dentistry in recent times is age estimation for various purposes such as orthodontics, forensic dentistry, human anthropology, and bioarchaeology. Assessment of calcification of dental tissue is another physiologic method for skeletal growth assessment.

Aims: This study aims to evaluate the correlation between dental calcification stages and skeletal maturity indicators and their application in age estimation purposes.

Methods: Tooth calcification was assessed by Demirjian's method and hand-wrist assessment was done by Fishman’s method. Spearman's rank-order correlation coefficient was applied to measure the association between skeletal maturational indicators and dental calcification stages of individual teeth, and the statistical significance of the correlation was tested.

Results: Spearman's significant coefficients for canine, first premolar, second premolar, and molar are 0.11, 0.09, 0.09, and 0.13, respectively, which are not significant.

Conclusion: Fishman's method of hand-wrist radiograph assessment is quite accurate as a maturity indicator but its association with dental calcification stages cannot be established.

## Introduction

Age estimation has become an important part of pediatric dentistry, orthodontics, forensic dentistry, human anthropology, and bioarchaeology. Different biological systems have their own unique indices for measuring children's development, and they include indices for sexual and somatic maturity as well as dental and skeletal maturation [1,2]. A patient's skeletal age is an important diagnostic and treatment planning tool because it provides insight into the likelihood that a certain therapy will be effective. However, genetic and ethnic variation as well as environmental factors affect the maturational time of the developing person [3].

In comparison to chronological age, physiologic maturity is more dependable and stable. Somatic, sexual, skeletal, or dental maturity may all be used to assess this stage of development [4]. Also, a single examination may be used to make a forecast based on valid relationships [5]. This technique has been used to evaluate skeletal development by examining bone changes in the hand and wrist. It is perfectly safe to take these radiographs because of the low effective dosage of 0.0001-0.1 mSV that is obtained during each exposure. Taking a two-minute transatlantic trip as an example, this dosage is equivalent to less than 20 minutes of natural background radiation [6,7]. The calcification of tooth tissue is another physiologic approach to assessing skeletal development. Because of its minimal variability, this approach seems to be dependable. It is the least impacted by endocrine, systemic, or other elements that influence the eruptive process of teeth [3,8]. This system is an intrinsic element of the human body, which means it should be investigated in tandem with other indications of physiological maturity, such as bone age, menarche, and height [9]. When dental and skeletal maturity signs are correlated, they may be used to estimate an individual's age. Consequently, the current work is focused on determining the link between dental calcification stages and skeletal maturity markers and their use in age assessment.

## Materials and methods

There were 120 hand-wrist radiographs and orthopantomographic (OPG) images of children aged eight to 14 years in this research. It was decided to only include patients who did not have any abnormalities in their facial features or histories of damage to their hands or wrists. Patients who have received orthodontic treatment or extractions were not included in the research. The ethical approval was taken from Vyas Dental College Ethical Committee (approval number: REF 23/12/14).

**Figure 1:**
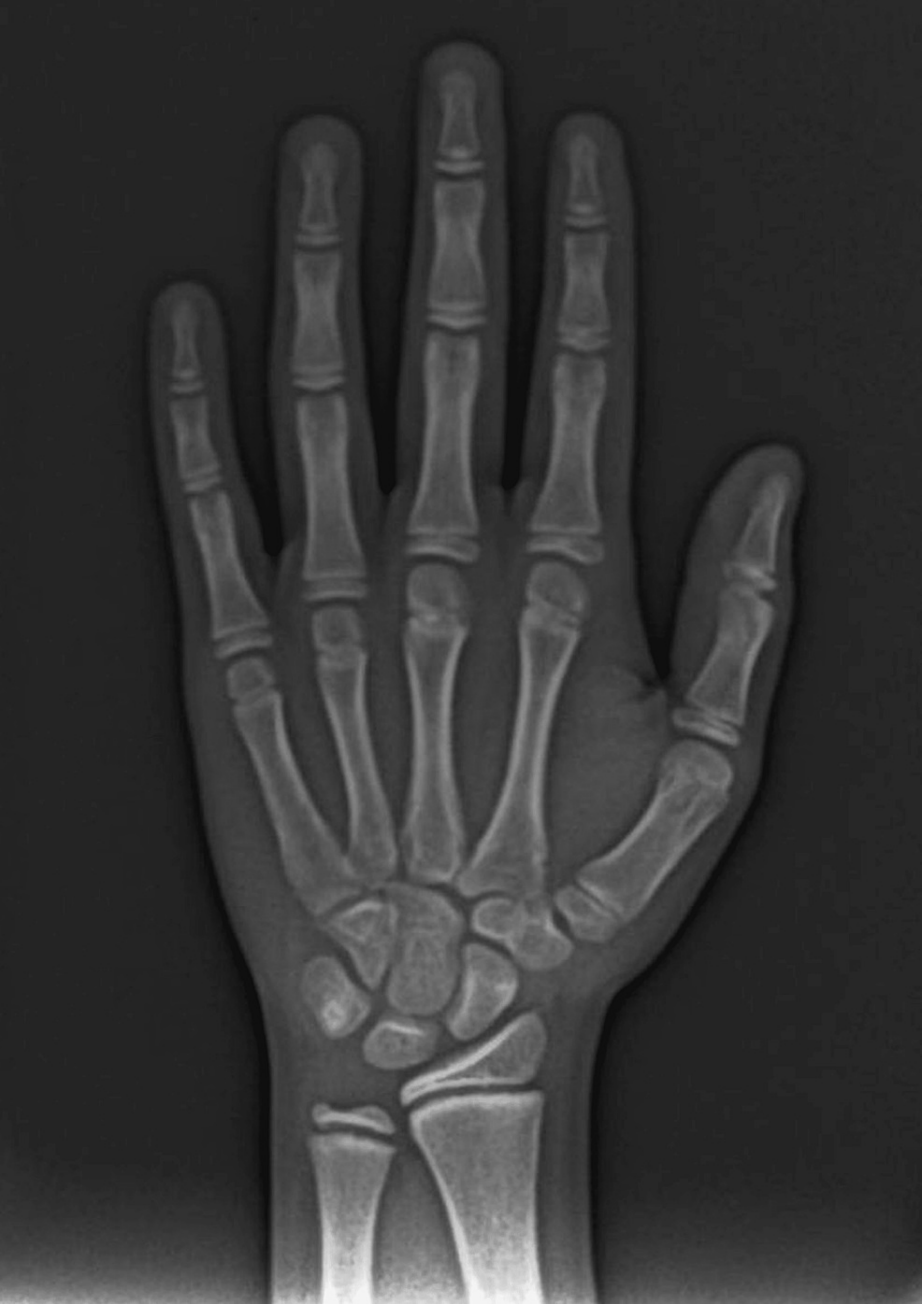
Hand-wrist radiograph

There were eight phases, A to H, in the approach described by Demirjian and colleagues, in which each tooth was allocated a number from A to H [10]. A hand-wrist radiograph is used to evaluate skeletal development in accordance with Fishman's technique. The four hardening phases are as follows: epiphyseal extension on selected phalanges, the solidification of the thumb's adductor sesamoid, the "covering" of selected epiphyses over their diaphyses, and the combining of selected epiphyses and diaphyses. However, the accompanying hardening choice is not a certain conclusion. The stages of evaluation are as follows: MP3: the middle phalanx of the third finger; the epiphysis is equal to its diaphysis. S stage: the first mineralization of the ulnar sesamoid bone. MP3cap: the middle phalanx of the third finger; the diaphysis is capped by epiphysis. DP3u: the distal phalanx of the third finger; complete epiphyseal union takes place. MP3u: the middle phalanx of the third finger; complete epiphyseal union takes place (Figure 1).

Statistical analysis was done to determine the correlation between skeletal maturational indicators and the dental calcification stages of individual teeth. The Spearman's position request correlation coefficient was used. The rate dispersion of the stages of calcification for each tooth was established to focus on the links between the phase of tooth mineralization and the phase of skeletal growth.

## Results

There is a strong correlation between tooth calcification phases and skeletal maturity stages, as illustrated in Tables 1-4.

**Table 1:** Hand-wrist maturation assessment of the canine

Sex				Canine				Total
				E	F	G	H	
Female	Hand-wrist	MP3 stage	n	0	20	9	6	35
			%	0.00	57.10	25.70	17.10	100.00
		MP3cap	n	0	5	4	5	14
			%	0.00	35.70	28.60	35.70	100.00
		MP3u	n	0	2	3	2	7
			%	0.00	28.60	42.90	28.60	100.00
		S stage	n	0	3	1	0	4
			%	0.00	75.00	25.00	0.00	100.00
	Total		n	0	30	17	13	60
			%	0.00	50.00	28.30	21.70	100.00
Male	Hand-wrist	MP3 stage	n	1	19	6	2	28
			%	3.60	67.90	21.40	7.10	100.00
		MP3cap	n	1	8	4	3	16
			%	6.30	50.00	25.00	18.80	100.00
		MP3u	n	0	6	2	0	8
			%	0.00	75.00	25.00	0.00	100.00
		S stage	n	0	6	2	0	8
			%	0.00	75.00	25.00	0.00	100.00
	Total		n	2	39	14	5	60
			%	3.30	65.00	23.30	8.30	100.00

**Table 2:** Hand-wrist maturation assessment of the first premolar

Sex				1st premolar					Total
				D	E	F	G	H	
Female	Hand-wrist	MP3 stage	n	0	6	15	6	8	35
			%	0.00	17.10	42.90	17.10	22.90	100.00
		MP3cap	n	0	1	5	3	5	14
			%	0.00	7.10	35.70	21.40	35.70	100.00
		MP3u	n	0	1	2	2	2	7
			%	0.00	14.30	28.60	28.60	28.60	100.00
		S stage	n	0	0	3	1	0	4
			%	0.00	0.00	75.00	25.00	0.00	100.00
	Total		n	0	8	25	12	15	60
			%	0.00	13.30	41.70	20.00	25.00	100.00
Male	Hand-wrist	MP3 stage	n	1	4	13	8	2	28
			%	3.60	14.30	46.40	28.60	7.10	100.00
		MP3cap	n	1	1	5	7	2	16
			%	6.30	6.30	31.30	43.80	12.50	100.00
		MP3u	n	0	1	5	2	0	8
			%	0.00	12.50	62.50	25.00	0.00	100.00
		S stage	n	0	0	6	1	1	8
			%	0.00	0.00	75.00	12.50	12.50	100.00
	Total		n	2	6	29	18	5	60
			%	3.30	10.00	48.30	30.00	8.30	100.00

**Table 3:** Hand-wrist maturation assessment of the second premolar

Hand-wrist * 2nd premolar									
Sex				2nd premolar					Total
				D	E	F	G	H	
Female	Hand-wrist	MP3 stage	n	1	7	14	7	6	35
			%	2.90	20.00	40.00	20.00	17.10	100.00
		MP3cap	n	1	0	5	3	5	14
			%	7.10	0.00	35.70	21.40	35.70	100.00
		MP3u	n	0	2	1	2	2	7
			%	0.00	28.60	14.30	28.60	28.60	100.00
		S stage	n	0	0	3	1	0	4
			%	0.00	0.00	75.00	25.00	0.00	100.00
	Total		n	2	9	23	13	13	60
			%	3.30	15.00	38.30	21.70	21.70	100.00
Male	Hand-wrist	MP3 stage	n	1	8	8	10	1	28
			%	3.60	28.60	28.60	35.70	3.60	100.00
		MP3cap	n	1	3	3	7	2	16
			%	6.30	18.80	18.80	43.80	12.50	100.00
		MP3u	n	0	2	4	2	0	8
			%	0.00	25.00	50.00	25.00	0.00	100.00
		S stage	n	0	3	3	1	1	8
			%	0.00	37.50	37.50	12.50	12.50	100.00
	Total		n	2	16	18	20	4	60
			%	3.30	26.70	30.00	33.30	6.70	100.00

**Table 4:** Hand-wrist maturation assessment of the second molar

Sex				2nd molar					Total
				D	E	F	G	H	
Female	Hand-wrist	MP3 stage	n	2	13	8	11	1	35
			%	5.70	37.10	22.90	31.40	2.90	100.00
		MP3cap	n	1	2	3	7	1	14
			%	7.10	14.30	21.40	50.00	7.10	100.00
		MP3u	n	1	1	1	4	0	7
			%	14.30	14.30	14.30	57.10	0.00	100.00
		S stage	n	0	1	3	0	0	4
			%	0.00	25.00	75.00	0.00	0.00	100.00
	Total		n	4	17	15	22	2	60
			%	6.70	28.30	25.00	36.70	3.30	100.00
Male	Hand-wrist	MP3 stage	n	7	6	8	7	0	28
			%	25.00	21.40	28.60	25.00	0.00	100.00
		MP3cap	n	1	3	5	7	0	16
			%	6.30	18.80	31.30	43.80	0.00	100.00
		MP3u	n	2	2	2	2	0	8
			%	25.00	25.00	25.00	25.00	0.00	100.00
		S stage	n	0	3	4	1	0	8
			%	0.00	37.50	50.00	12.50	0.00	100.00
	Total		n	10	14	19	17	0	60
			%	16.70	23.30	31.70	28.30	0.00	100.00

When comparing canine, first, and second premolar calcification phases with those of the wrists' hands, this research found an unbalanced distribution. The canine F stage was the most common in both males and females in the MP3 stage. The S stage of canine development was not uniformly distributed. Females' MP3cap stage second molar G and canine and first premolar H stages were both 35% G and 35% H, respectively. In the canine and first premolar, males had a dispersed distribution in the F and G stages. A majority of the canine's first, second, and third premolars were found in the F and G stages in both male and female MP3u stage canines.

There is no statistical significance in the coefficients of Spearman's test for the canine, first premolar, second premolar, and molar, as shown in Table 5.

**Table 5:** Spearman's rank order correlation coefficients between the developmental stages of the hand and wrist bones and the developmental stages of the four individual teeth

Correlations						
			Canine right	First premolar right	Second premolar right	Second molar right
Spearman's rho	Hand-wrist	r-value	0.118	0.097	0.099	0.135
		p-value	0.201	0.291	0.284	0.141
		N	120	120	120	120

## Discussion

The notion of physiological age was developed as a way to measure the progress of a child's growth or maturity because of the disparities in the developmental stages of distinct biological systems. Child dentition may be utilized as one of the skeletal approaches for physiological age assessment since children of the same chronological age have a wide range of development [11]. The Demirjian technique is extremely accurate and has a strong correlation with chronological age [12].

As a continuous and progressive process that can be monitored radiographically, tooth creation has been a more extensively utilized method of monitoring dental maturation than tooth eruption since most teeth can be assessed at each examination. The dental age of a person may be determined by combining information about the development phases of numerous teeth [13]. The Fishman approach has long been a go-to tool for determining skeletal maturity. Many researchers have documented the use of hand-wrist radiographs as an indicator for assessing skeletal maturity based on the timing and sequencing of the emergence of carpal bones and specific ossification processes [14,15]. This approach to skeletal maturation was also used by Mohammed et al. in their research of the South Indian population as a reliable means of determining an individual's mean age [16].

Canine, premolar, and molar calcification stages were found to be unevenly distributed in comparison to the hand and wrist stages in this investigation. Saglam and Gazilerli's research also found a low correlation coefficient between skeletal and oral maturity indices [17]. The Spearman's significant coefficients for canine, first premolar, second premolar, and molar are 0.20, 0.29, 0.28, and 0.14, respectively, which are not statistically significant. Although Rai et al. found no association between dental and skeletal maturation, they found high correlations between the two [18]. Additionally, investigations by Kiran et al. and Günen Yılmaz et al. found an association between oral calcification stages and skeletal maturity indices [19,20].

## Conclusions

Fishman's method of hand-wrist radiograph assessment is quite accurate as a maturity indicator but its association with dental calcification stages cannot be established with such a small sample size. Therefore, the application of Demirjian’s method for age assessment is doubtful and further studies with a larger population are required.
